# The Mallet

**Published:** 1863-10

**Authors:** 


### THE MALLET—NEW THINGS.

We are still very frequently asked, "Do you use the mallet yet as much as ever?" We answer yes, and we have answered it so often, that it's an old story. The mallet has become an institution, with us at least, and when we cease using the mallet we expect to quit the practice of dentistry.

We use it because we can make better fillings, can consolidate gold better and adapt it better, can manipulate with far more precision; it is more acceptable to the patient than hand pressure, because easier; by it, there is no liability of injuring the surrounding tissues, and consequently far less irritating to the patient: and again, it renders an operation far less fatiguing and laborious to the operator.

We have recently heard of three or four attempts, by as many different parties, to construct an automatic mallet, one of these, at least, is patented, or a caveat filed therefor, (the patent fever still rages.) We do not pretend to venture an opinion as to what may be brought forth while the matter is still in embryo. We shall probably, ere long, have a deliverance on this subject.

